# Comments on “dynamic ^18^F‐FDG PET/CT can predict the major pathological response to neoadjuvant immunotherapy in non‐small cell lung cancer”

**DOI:** 10.1111/1759-7714.14710

**Published:** 2022-10-26

**Authors:** Shan‐Ying Wang, Yu‐Chien Shiau, Chen‐Xiong Hsu

**Affiliations:** ^1^ Department of Nuclear Medicine Far Eastern Memorial Hospital New Taipei City Taiwan; ^2^ Department of Biomedical Imaging and Radiological Sciences National Yang Ming Chiao Tung University Taipei Taiwan; ^3^ Division of Radiation Oncology, Department of Radiology Far Eastern Memorial Hospital New Taipei City Taiwan

**Keywords:** 18F‐FDG PET/CT, volume‐based metabolic parameters, SUV, metabolic tumor volume, total lesion glycolysis

To the Editor,

We appreciate the insights of Chen et al. with regard to the real‐world retrospective study to identify the association between the dynamic changes in ^18^F‐labeled fluoro‐2‐deoxyglucose positron emission tomography (^18^F‐FDG PET)/computed tomography (CT) and the major pathological responses (MPR) in 44 stage II–III non‐small cell lung cancer patients receiving neoadjuvant immunotherapy and subsequent surgery.^1^

We agree that ^18^F‐FDG PET/CT serves as a decisive tool in detection, staging, and response assessment in cancer patients. In the article recently published by Chen et al. in *Thoracic Cancer*, the dynamic longest dimension and dynamic changes of maximum SUV (SUVmax) showed significant differences between the MPR (*n* = 28) and non‐MPR (*n* = 16) groups. Moreover, the decline in SUVmax was an independent factor for major pathological responses, and the dynamic SUVmax decreasing ≥60% might be the ideal threshold on receiver‐operating characteristic analysis.^1^ However, non‐small cell lung cancer is a highly heterogeneous disease. The SUVmax of the tumor indicates a semi‐quantitative estimate of the highest ^18^F‐FDG uptake in a single voxel of a single mass which cannot be representative of the dynamic metabolic changes of the whole disease,^2^ whereas the metabolic tumor volume (MTV) and total lesion glycolysis (TLG) provide not only the total metabolic burden of primary tumors but also lymph nodes and metastatic lesions.^3^, ^4^, ^5^

Furthermore, a number of studies and review articles have emphasized the fundamental role of the three‐dimensional parameters derived from ^18^F‐FDG PET images including the MTV and TLG which provide better predictive and prognostic values for survival of non‐small cell lung cancer patients as compared with SUVmax.^6^, ^7^ Given these considerations, the essential role of volume‐based metabolic parameters in the modern era of immunotherapy of lung cancer should be carefully considered for the future work of Chen and colleagues.

## CONFLICT OF INTEREST

The authors declare no conflict of interest.
